# METTL14 suppresses cancer stem cell phenotype of colorectal cancer via regulating of β-catenin/NANOG

**DOI:** 10.7150/jca.82158

**Published:** 2023-05-15

**Authors:** Chun-Lei Sun, Jiong Chen, Zhi-Wei Xing, Gong-Shai Tao

**Affiliations:** 1 Cheeloo College of Medicine, Shandong University, Jinan, China.; 2 The First Affiliated Hospital of USTC, Division of Life Sciences and Medicine, University of Science and Technology of China, Hefei, China, 230001.

**Keywords:** Colorectal cancer, cancer stem cell phenotype, m6A, METTL14, β-catenin, NANOG

## Abstract

Cancer stem cell (CSC) characteristic contributes to tumor malignancy and progression. The role of N6-methyladenosine (m6A) modification in CSC characteristic is largely unknown. In this study, we found that m6A methyltransferase METTL14 was downregulated in colorectal cancer (CRC) and negatively correlated with the poor prognosis of CRC patients. Overexpression of METTL14 inhibited CSC characteristic, while knockdown of METTL14 promoted this characteristic. Through screening, NANOG was identified as the downstream of METTL14. Mechanically, we demonstrated that METTL14 inhibited cancer stem cell characteristic by regulating β-catenin. Collectively, our findings suggested that METTL16/β-catenin /NANOG axis might be promising therapeutic targets for CRC.

## Introduction

As one of the most common malignancies, colorectal cancer (CRC) ranks the third among leading causes of cancer deaths worldwide 1. Despite great advance in surgery and neoadjuvant therapy has enhanced the survival rate of CRC in the past few decades, the prognosis of CRC patients remains unsatisfactory due to tumor metastasis and recurrence 2. Thus, it is urgent to reveal the molecular mechanism of CRC metastasis and recurrence, which will be meaningful for development of potential therapeutic therapy for CRC.

Compelling evidence has revealed that malignant tumors frequently display a hierarchical organization, whose apex is occupied by a subset of cancer cells named cancer stem cells (CSCs) due to the capacity to self-renew and differentiate into the heterogeneous lineages of cancer cells 3, 4. CSCs have been proved to exist in a variety of cancers, such as CRC and breast cancer, where they are recognized to promote tumorigenesis and are responsible for cancer recurrence, metastasis, and chemoresistance 5-7. Moreover, CSCs have been identified as the major source of tumor heterogeneity, which in turn contributes to tumor recurrence, dissemination, and therapy resistance 8-11. Several pluripotent makers, including NANOG, SOX2, BMI-1, and OCT-4, play an important role in maintaining the stemness and differentiative potential of CSCs 12-15. Among them, transcriptional factor NANOG is known to high expressed in various cancer cells and acts as an essential modulator of CSCs. In addition, Abnormal activation of critical signaling pathways have been implicated in sustaining the CSC aggressive characteristic 16, 17, such as Wnt signal 18, 19. Targeting these key regulators and signaling pathways may be a promising strategy to eliminate CSCs mediated-tumor progression.

N6-methyladnosine (m6A) methylation, a mRNA chemically modification analogous to histones and DNA, extensively exists in eukaryotic mRNAs 20. As a dynamic and reversible modification, m6A can be triggered by methyltransferases (also named writer), including METTL3, METTL14, WTAP, and KIAA1429, and can be eliminated by demethylases (also named eraser) consists of FTO and ALKBH5 21-23. Furthermore, RNA binding proteins play an indispensable role in m6A methylation by identifying and binding m6A motif, such as YTHDF1/2/3, IGF2BP1/2/3, and HNRNPC 24-26. Through the synergistic action of writers, erasers, and readers, m6A modification regulates the fate of mRNA in splicing, translation, decay, stability, and nuclear export 27. Increasing researches demonstrated the crucial effects of m6A modification in normal development and diverse disease 28, 29.

As a main m6A methyltransferase, METTL14 has been proved to be crucial for tumorigenesis and development in hepatocellular carcinoma, gastric cancer, leukemia, renal cancer, bladder cancer, CRC and so on 21, 30. However, the role and molecular mechanism of METTL14 in CSCs progression is little known. In this study, we first found that METTL14 can inhibit the CSC phenotype of CRC and revealed the underlying molecular mechanism. Our findings indicated that METTL14 might be a potential therapeutic target for CRC.

## Materials and Methods

### Cell culture

Colorectal cancer cell lines HCT8, HCT15, HCT116, HT29, SW480, SW620, Lovo, RKO, and colon epithelial cell lines NCM460 were all purchased from American Type Culture Collection. HCT116, HT29 cells were cultured in McCoys'5A (Hyclone), NCM460, SW480, SW620, RKO cells were cultured in DMEM (Gibco), and HCT8, HCT15 cells were cultured in RPMI-1640 (Hyclone) respectively. All cell lines were grown in mediums supplemented with 10% fetal bovine serum (BI) and cultured in 5% CO2 at 37 °C.

### Mammosphere formation assay

Cells were seeded in 12-well ultralow attachment plates (Corning) at a density of 3000 cells/ml and cultured for 10 days in mammosphere culture, which consists of serum-free DMEM/F12 (Invitrogen), 1:100 B27 (Invitrogen), 20 ng/mL EGF (Invitrogen), 20 ng/mL bFGF (Invitrogen), 5 g/mL insulin, 1 g/mL hydrocortisone and 1% antibioticantimycotic. Fresh medium was added every 3 days. The number of mammospheres (>30 μm) was counted under an upright microscope.

### Gene over-expression and RNA interference

pcDNA3.1-METTL14 (METTL14-OE) and empty Vector were obtained from Hanbio Biotechnology Co.Ltd (Shanghai). Small interfering RNAs (siRNA) targeted METTL14, NANOG, β-catenin and corresponding negative controls (siNC) were purchased from GenePharma Company (Shanghai). According to the manufacturer's protocols, cells were transfected with 2 μg plasmid vector or 100 pmol siRNA oligomer mixed with 5μl lipofectamine 2000 reagent in serum free medium. Medium was changed to complete culture medium 6 h later, and the cells were incubated for another 24-48 h before harvest.

### Quantitative real-time PCR

Total RNA of cells was extracted using TRIzol Regent (Invitrogen, Carlsbad, CA, USA) according to the instructions and was reverse transcribed to cDNA with Prime Script RT master Mix (TakaRa, Tokyo, Japan). Every 500 ng cDNA was mixed with specific primers and SYBR Premix Ex Taq II (TakaRa, Tokyo, Japan) regent and performed to quantitative real-time PCR. 2-ΔΔCt method was used to calculate each group relative mRNA amount by normalizing to GAPDH. The primer sequences used in this study were listed in Table 1.

### Western blotting

Cells were lysed with RIPA lysis buffer (Solarbio) on ice for 15 mins and centrifugated at 10000 rpm/min at 4°C for 30 mins. The concentration of protein from cell supernatant was quantified using the BCA regent. The equal amount of protein samples was loaded on SDS-PAGE and then transferred to PVDF membrane (Beyotime), blocked with 5% skimmed milk (Solarbio) at room temperature for 2 h and incubated with primary antibody at 4 °C overnight. Primary antibodies were listed as: anti-METTL14 (HPA038002, SigmaAldrich), anti-NANOG (4893, CST), anti-β-catenin (sc-7963, Santa Curz). Afterwards, the PVDF membrane was washed for 3 times with PBS, incubated with primary antibody at room temperature for 1.5 h and the chemiluminescence was detected by Pierce™ ECL Western Blotting Substrate (32106, Thermo Fisher Scientific).

### Database analysis

Expression levels of METTL14 in cancer tissues and normal tissues of colorectal cancer were obtained from TCGA (The Cancer Genome Atlas) database. METTL14 expression in colorectal cancer from GEO database (GSE41657, GSE44076) was analyzed. Kaplan-Meier was used to assess the prognostic value of METTL14 expression in patients with colorectal cancers from TCGA database.

### Statistical analysis

All data were statistically analyzed with SPSS 20.0 and GraphPad Prism 5.0. P < 0.05 was identified as statistical significance. Two-tailed unpaired Student's t-test was used to analyze data from two groups. One-Way ANOVA was used followed by Bonferroni test to analyze multiple comparisons. For survival analysis, Kaplan-Meier analysis was employed.

## Results

### METTL14 is downregulated in CRC patients and correlates with poor prognosis

**Figure 1:**
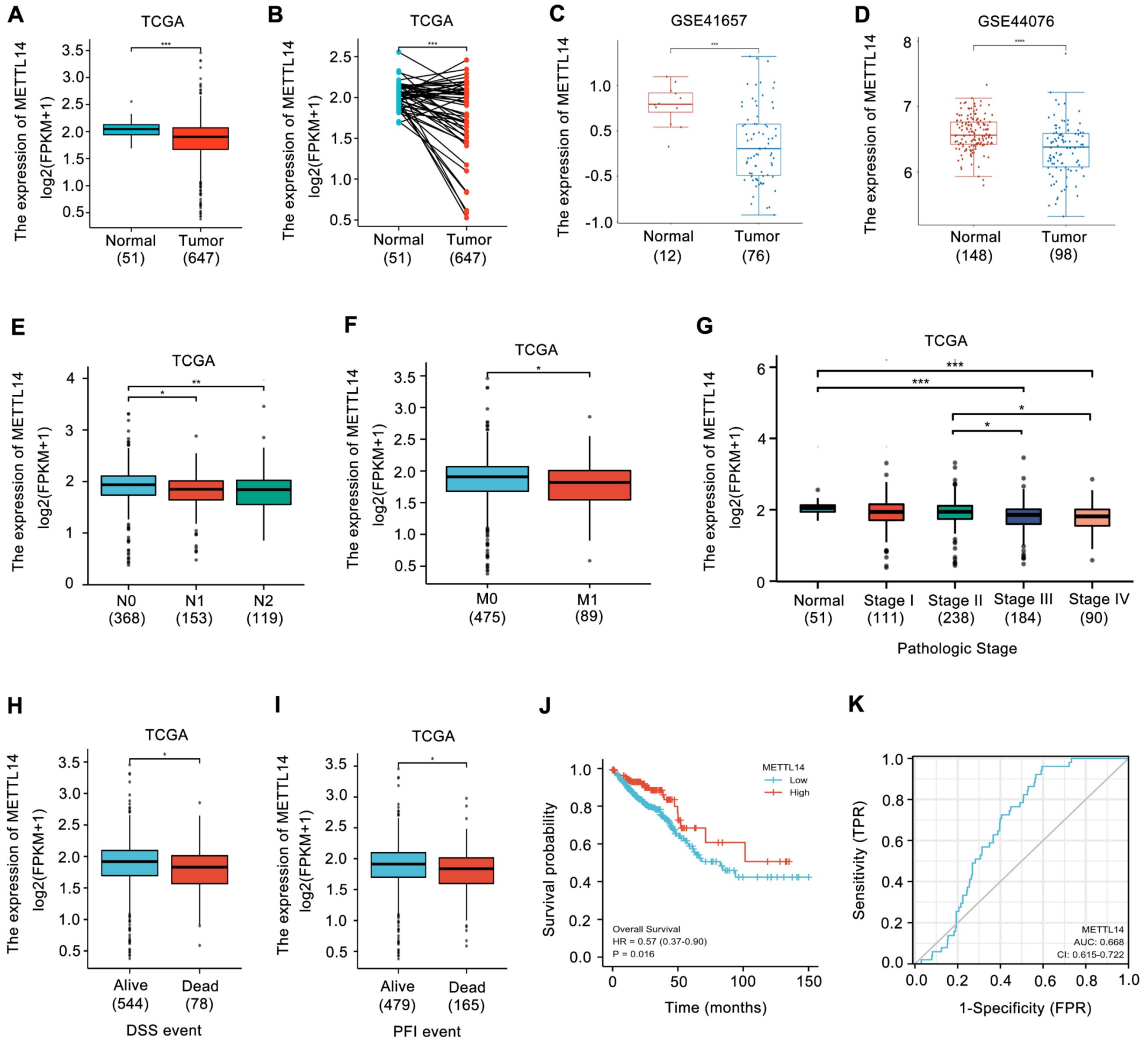
** METTL14 is downregulated in CRC and correlated with poor survival.** (A-B) METTL14 gene expression in the TCGA CRC cohort. (C-D) METTL14 gene expression in the GSE41657 (C) and GSE44076 (D) CRC cohorts. (E-G) Association of METTL16 mRNA expression with lymph node metastasis (E), distant metastasis (F) and pathologic stage (G) in CRC patients in TCGA database. N0: no lymph node metastasis; N1: nearby lymph node metastasis; N2: distant lymph node metastasis; M0: no distant metastasis; M1: distant metastasis. (H-I) Association of METTL16 mRNA expression with PFI (Progression-Free Interval) (H) and DSS (Disease Free Survival) (I) in CRC patients in TCGA database. (J) Kaplan-Meier survival curves of OS based on METTL14 expression in CRC patients in TCGA database. (K) The ROC curve of METTL16 in predicting tumorigenesis of CRC. *P<0.05, **P<0.01, ***P<0.001.

METTL14 expression in CRC was first analyzed. Upon analyzing the TCGA database, the expression of METTL14 was found to be decreased in CRC samples (Fig. 1A, B). Similarly, the results from GEO database also showed that METTL14 was distinctly downregulated in CRC tissues compared with normal tissues (Fig. 1C, D). Moreover, decreased METTL14 expression was tightly associated with tumor lymph node metastasis (Fig. 1E), distant metastasis (Fig. 1F), and pathologic stage (Fig. 1G). Survival analysis from TCGA database showed that decreased METTL14 expression corelated with worse survival (Fig. 1H, I). Kaplan-Meier survival analysis further demonstrated that CRC patients with lower METTL14 expression had a poor overall survival (Fig. 1J). Importantly, the ROC curve found that METTL14 expression could be a predictor of CRC tumorigenesis (Fig. 1K). Taken together, these results suggested that METTL14 expression was downregulated in CRC and might be associated with tumor progression.

### METTL14 inhibits CRC stem cell phenotype

**Figure 2:**
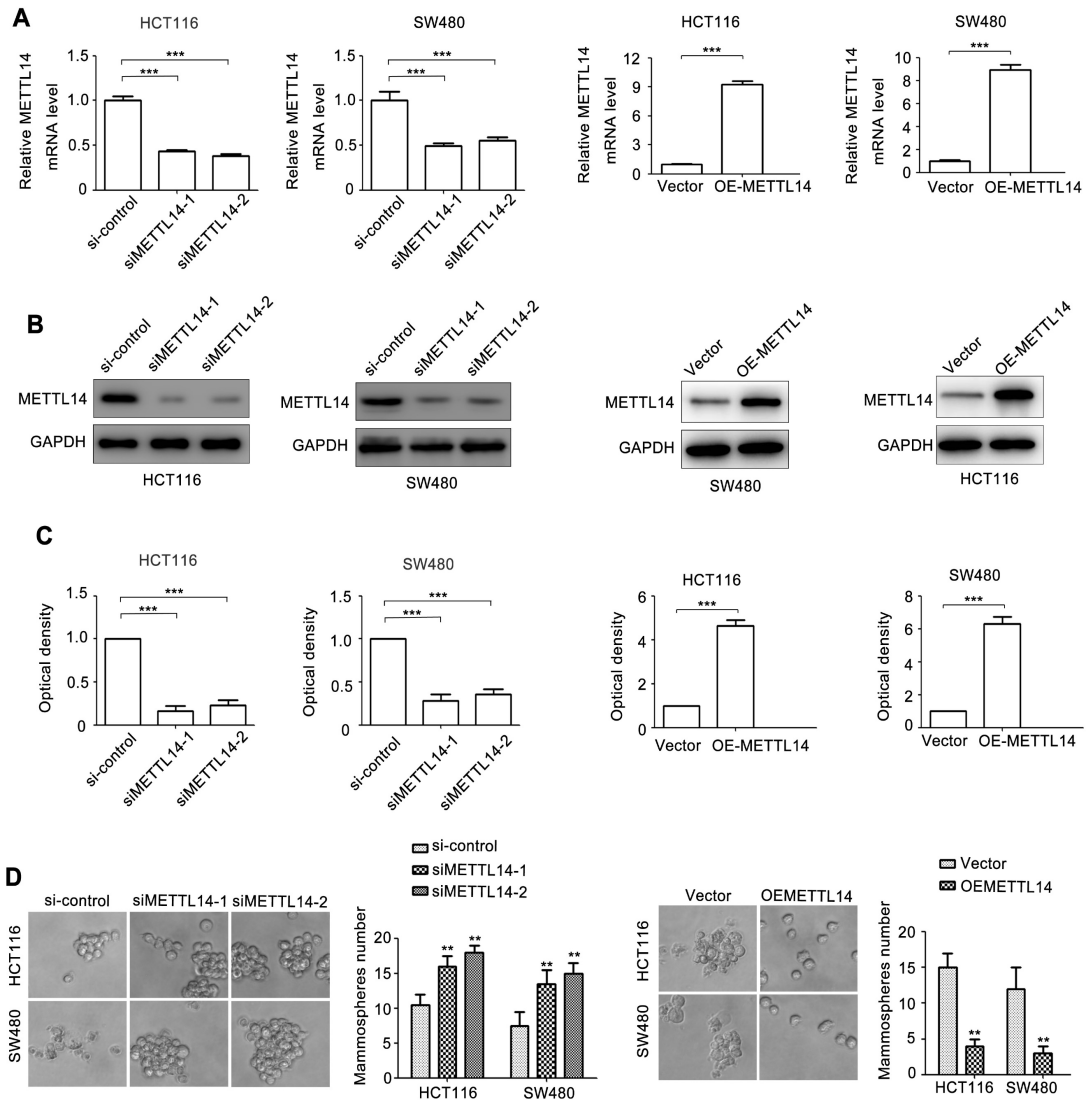
** METTL14 inhibits CRC stem cell phenotype.** (A) The knockdown and overexpression efficiency of METTL14 in SW480 and HCT116 cells were confirmed by qRT-PCR. (B-C) The knockdown and overexpression efficiency of METTL14 in SW480 and HCT116 cells were confirmed by western blot. (D) The effect of knockdown and overexpression of METTL14 on mammosphere formation of SW480 and HCT116 cells. **P<0.01, ***P<0.001.

To investigate the role of METTL14 in CRC, two siRNAs of METTL14 (siMETTL14-1, siMETTL14-2) were used to knocked down METTL14 expression. Meanwhile, an overexpressed vector pcDNA3.1-METTL14 was used to elevated METTL14 expression in CRC. As showed in Fig. 2A, HCT116 and SW480 cells transfected with siMETTL14-1 or siMETTL14-2 significantly inhibited METTL14 mRNA expression. On the contrary, pcDNA3.1-METTL14 transfection obviously upregulated METTL14 mRNA expression in HCT116 and SW480 cells. Besides mRNA, METTL14 protein expression in HCT116 and SW480 cells transfected with siMETTL14-1, siMETTL14-2 or pcDNA3.1-METTL14 displayed a similar effect (Fig. 2B, C). Previous studies showed that METTL14 could inhibits colorectal cancer metastasis and proliferation. Since tumor metastasis and proliferation are closely associated with cancer stem cell properties, we investigate the effect of METTL14 on microsphere formation ability, which indicate cancer stem cell properties. Interestingly, we found that knockdown of METTL14 promoted the mammosphere formation ability of HCT116 and SW480 cells, while METTL14 overexpression suppressed CRC cells mammosphere formation ability. These results stem cell phenotype proved that METTL14 could inhibit the stem cell phenotype of CRC cells.

### METTL14 inhibits NANOG expression in CRC cells

**Figure 3:**
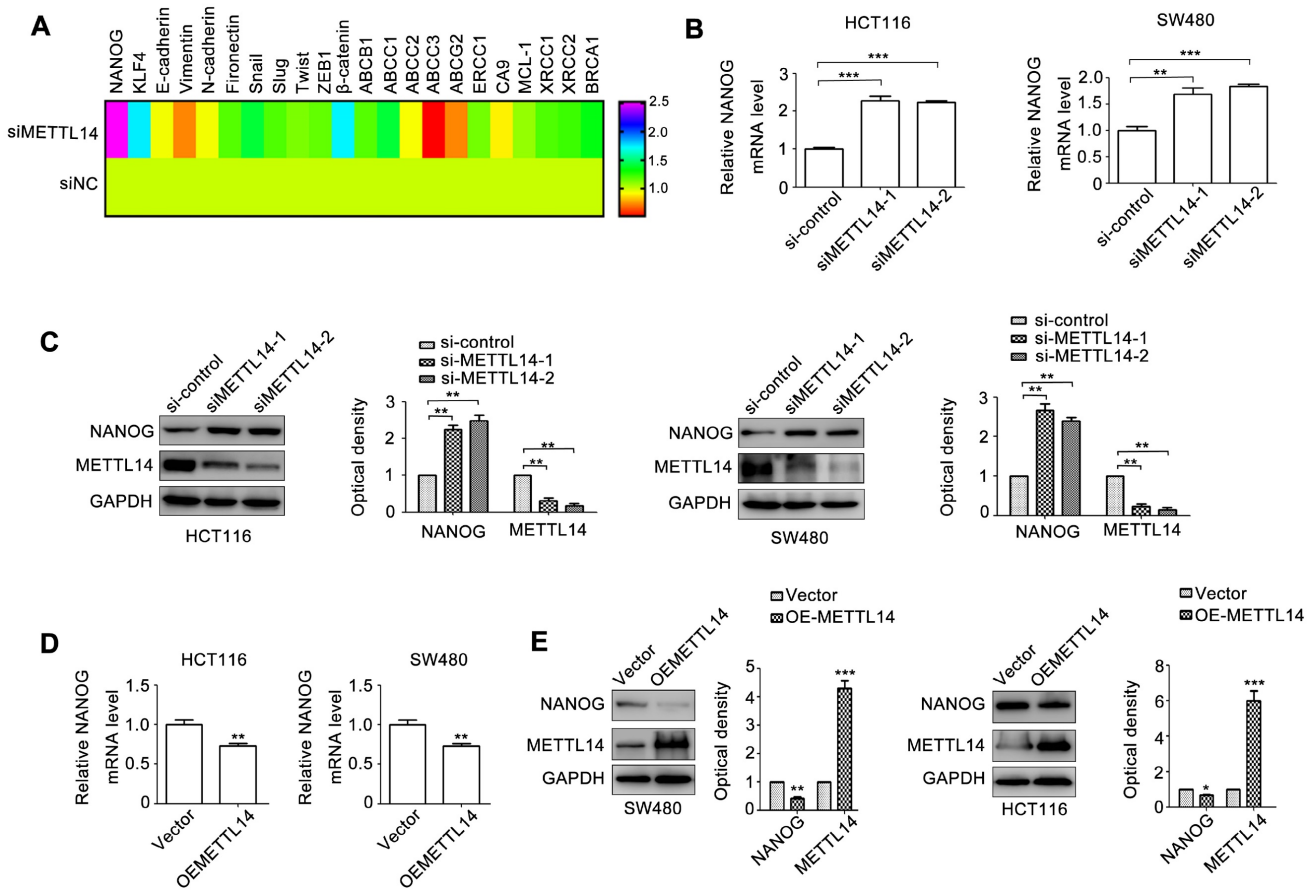
** METTL14 inhibits NANOG expression in CRC cells.** (A) A set of oncogenes gene expression in HCT116 with METTL14 knockdown were detected by qRT-PCR. (B) The effect of METTL14 knockdown on NANOG gene expression in SW480 and HCT116 cells were detected by qRT-PCR. (C) The effect of METTL14 knockdown on NANOG protein expression in SW480 and HCT116 cells were detected by western blot. (D) The effect of METTL14 overexpression on NANOG gene expression in SW480 and HCT116 cells were detected by qRT-PCR. (E) The effect of METTL14 overexpression on NANOG protein expression in SW480 and HCT116 cells were detected by western blot. **P<0.01, ***P<0.001.

Cancer stem cell phenotype contributes to cancer progression 3. We next detected a set of molecules relative to cancer stem cell phenotype, metastasis, and chemoresistance. NANOG and KLF4 are transcriptional factors regulating cancer stem cell phenotype. E-cadherin, Vimentin, N-cadherin, Fibronectin, Snail, Slug, Twist, and ZEB1 are crucial biomarkers and regulators of EMT, an important initial step of tumor metastasis. ABCB1, ABCC1\2\3, ABCG2, ERCC1, XRCC1\2 can induce tumor chemo-resistance and radio-resistance. CA9, MCL-1, and BRCA1 can promote tumor anti-apoptosis. β-catenin is critical transcriptional factor controlling cancer progression. We next detected the effect of METTL14 on the 22 genes expression. As shown in Fig. 3A, among these molecules, NANOG expression displayed the most obvious change due to METTL14 knockdown. Through verifying, we demonstrated that METTL14 knockdown increased NANOG mRNA expression in SW480 and HCT116 cells (Fig. 3B). METTL14 loss also upregulated NANOG protein expression in SW480 and HCT116 cells (Fig. 3C). On the contrary, overexpression of METTL14 significantly decreased NANOG mRNA and protein expression.

### METTL14 inhibits β-catenin expression in CRC cells

**Figure 4:**
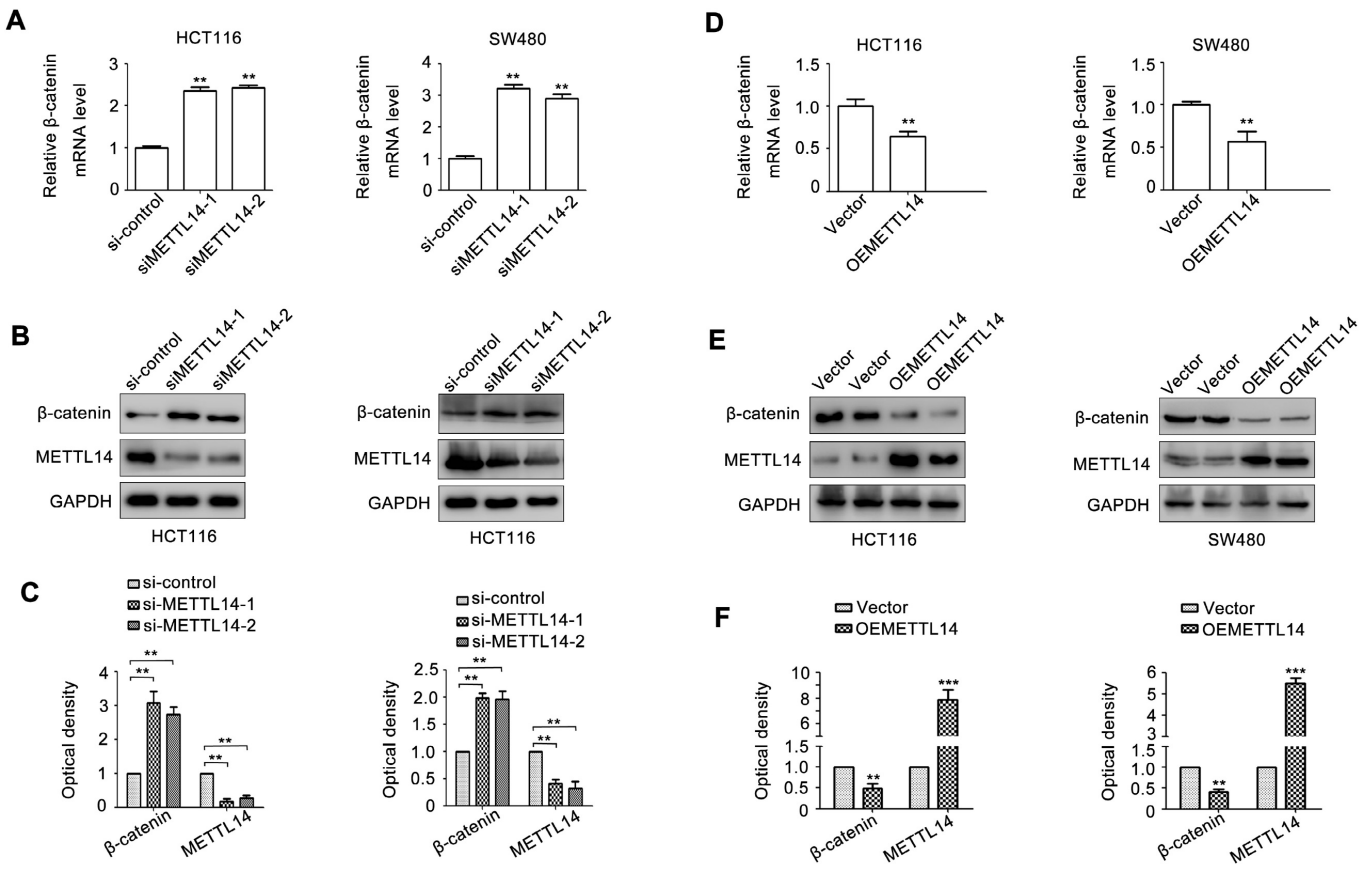
** METTL14 inhibits β-catenin expression in CRC cells.** (A) The effect of METTL14 knockdown on β-catenin gene expression in SW480 and HCT116 cells were detected by qRT-PCR. (B-C) The effect of METTL14 knockdown on β-catenin protein expression in SW480 and HCT116 cells were detected by western blot. (D) The effect of METTL14 overexpression on β-catenin gene expression in SW480 and HCT116 cells were detected by qRT-PCR. (E-F) The effect of METTL14 overexpression on β-catenin protein expression in SW480 and HCT116 cells were detected by western blot. **P<0.01, ***P<0.001.

Since β-catenin plays an important role in maintaining cancer stem cell phenotype 31. We next detect the effect of METTL14 on β-catenin expression. As shown in Fig. 4A, METTL14 knockdown significantly increased β-catenin mRNA expression in SW480 and HCT116 cells. METTL14 loss also upregulated β-catenin protein expression in SW480 and HCT116 cells (Fig. 4B, C). On the contrary, overexpression of METTL14 decreased β-catenin mRNA and protein expression (Fig. 4D-F). These results suggested that β-catenin might be involved in METTL14-mediated cancer stem cell phenotype in CRC cells.

### β-catenin plays an important role in METTL14-mediated stem cell phenotype

**Figure 5:**
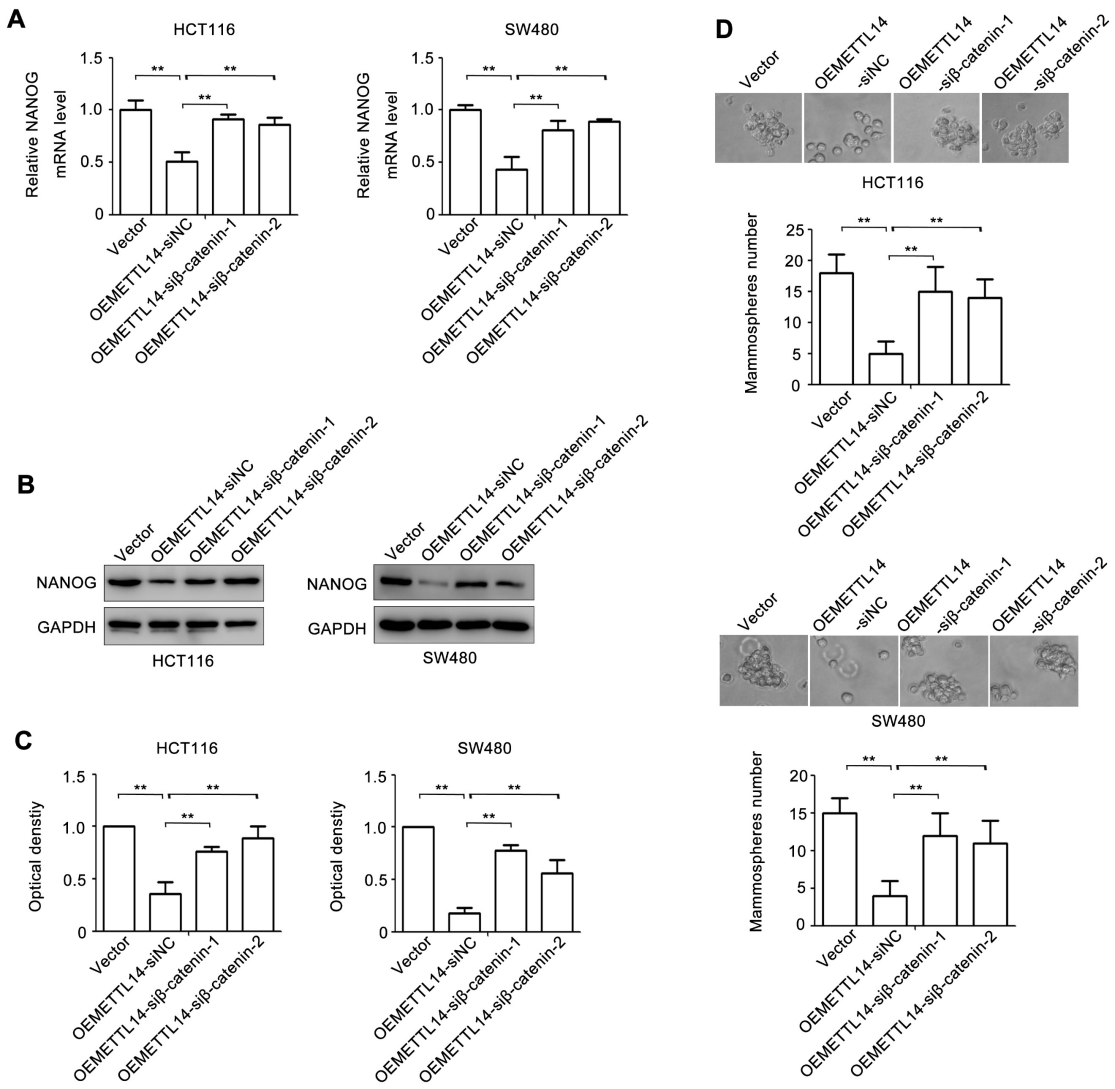
** β-catenin is involved in METTL14-inhibited stem cell phenotype.** (A) The effect of β-catenin knockdown on NANOG gene expression in METTL14 overexpressed SW480 and HCT116 cells were detected by qRT-PCR. (B-C) The effect of β-catenin knockdown on NANOG protein expression in METTL14 overexpressed SW480 and HCT116 cells were detected by western blot. (D) The effect of β-catenin knockdown on stem cell phenotype in METTL14 overexpressed SW480 and HCT116 cells were detected by mammosphere formation assay. **P<0.01.

To verify the role of β-catenin in METTL14-mediated stem cell phenotype of CRC, we knockdown β-catenin by using siRNA. The effect of β-catenin loss on NANOG expression was examined. As shown in Fig. 5A, overexpression of METTL14 decreased NANOG mRNA expression. However, knockdown of β-catenin in METTL14 overexpressed HCT116 and SW480 cells reversed METTL14-inhibited NANOG mRNA expression. The similar phenomenon was found at protein level. knockdown of β-catenin reversed METTL14-inhibited NANOG protein expression in HCT116 and SW480 cells (Fig. 5B, C). Next, we detected the effect ofβ-catenin loss on mammosphere formation. The results showed that β-catenin knockdown reversed METTL14-inhibited mammosphere formation ability in HCT116 and SW480 cells (Fig. 5D). Collectively, these results indicated that β-catenin played an important role in METTL14-mediated stem cell phenotype in CRC cells.

## Discussion

Besides histone and DNA, RNA also can be chemical modified. Up to now, there are over 100 kinds of posttranscriptional modification on RNA have been confirmed. Among them, m6A methylation is the most abundant RNA modification and accounts nearly 80% of the total RNA epigenetic regulation 29. Since the essential roles of m6A methylation in human physiological and pathological processes, it has been become an important research hotspot in recent five years. As a pivotal m6A methyltransferases, METTL14 has been reported to regulate various tumors progression, including CRC 21, 32. For example, METTL14 can suppresses CRC metastasis and proliferation via regulating different downstream targets, such as ARRDC4, SOX4, circORC5, miR-375, and lncRNA XIST 32-36. In addition, METTL14 elevates the response of CRC to programmed cell death-1 (PD-1) therapy and mediates chemoresistance of CRC to cetuximab 37, 38. However, the role and underlying molecular basis of METTL14 in CSCs progression is rarely known. Here, we showed that METTL14 can inhibit CSC phenotype of CRC. METTL14 expression is obviously decreased in CRC and closely corelates with prognosis of CRC patients. METTL14 silencing promotes CRC cells microsphere formation ability and increases pluripotent maker NANOG expression, whereas METTL14 overexpression has the opposite effects. Mechanistically, β-catenin mediates METTL14-inhibited microsphere formation ability and NANOG expression of CRC.

Increasing evidences demonstrated that m6A modulators could mediate CSC phenotype. The key m6A methyltransferases METTL3 affects CSC characteristic, proliferation, and apoptosis of multiple myeloma cells through stabilizing transcriptional factor YY1 mRNA and expediting primary-miR-27a-3p maturation in a m6A dependent manner 39. Chen G et.al found that demethylases ALKBH5 could promote pluripotent stem cell transcription factor SOX2 transcription via removing its mRNA methylation, thus sustaining the stemness and tumorigenicity potential of endometrial cancer stem cells 40. In addition, the reader YTHDF3 contributes to tumorigenicity of ocular melanoma CSCs via promoting CTNNB1 translation efficiency 41. YTHDF2 mediated-m6A modification on pluripotent maker OCT-4 strengthens CSC phenotype of liver cancer and induces tumor metastasis 42. In this study, we first showed that METTL14 could suppress stemness of CRC cells and increased NANOG expression. NAONG has been identified as one of the most critical transcriptional factors regulating pluripotent stem cell phenotype 43. It has been proved that NANOG plays a tumor-promoting role in various tumors development, including lung cancer, breast cancer, liver cancer, pancreatic cancer, CRC and so on 43, 44. In CRC, NANOG is significantly upregulated and associates with the poor prognosis of patients 45. A recent study revealed that hypoxia tumor microenvironment increased ALKBH5 expression, which eliminated m6A modified NANOG and enhanced its mRNA stability, thereby promoted breast cancer stem cell phenotype 46. Similarly, we demonstrated that NANOG played an important role in METTL14-inhibited stemness of CRC, suggesting that METTL14/NANOG axis may be a potential therapeutic target for CRC.

Wnt signaling pathway plays a crucial role in various tumors formation and progression 19. As the key downstream of Wnt signaling, transcriptional factor β-catenin exerts its function in maintaining CSC properties via transcriptionally regulating different targets 47. For example, Fu LC et.al showed that PD-L1 and β-catenin signaling make up a positive feedback loop to accelerate CSCs expansion and cancer progression by targeting putative stem cell marker LGR5 48. The activation of miRNA-146a/AKT/β-catenin signaling promotes CSC phenotype in oral squamous cell carcinoma by regulating CD24, a classical maker of CSC 49. Moreover, β-catenin mediates SOX2 induced-CSC phenotype, metastasis, and chemoresistance of CRC 50. In this study, we found that β-catenin is involved in METTL14-inhibited CSC properties and NANOG expression in CRC cells. Recently, emerging evidences indicated that β-catenin could be modified by m6A methylation. For example, m6A demethylase FTO upregulates β-catenin by removing the methylation modification of its mRNA and thus induces chemo-radiotherapy resistance of cervical squamous cell carcinoma 51. A recent study discovered that METTL3 controls the transcription, mRNA decay, translation efficiency, and sub-cellular localization of β-catenin relying on different reader proteins in a m6A dependent manner 52. Therefore, we speculated that METTL14 could directly regulate β-catenin expression in a m6A dependent manner. It worth to investigate the molecular mechanism of β-catenin expression regulated by METTL14 in the future study.

In summary, our findings suggest the tumor-suppressing role of METTL14 in CRC progression. METTL14 is downregulated in CRC and associates with the poor prognosis. Mechanistically, METTL14 inhibits CSC phenotype of CRC via regulating of β-catenin/NANOG, suggesting that METTL14/β-catenin/NANOG axis may be a potential therapeutic target for CRC.

## Figures and Tables

**Table 1:** Primers used in the RT-PCR assay.

Gene	Forward primer 5'-3'	Reverse primer 5'-3'
NANOG	CAAAGGCAAACAACCCACTT	TCTGCTGGAGGCTGAGGTAT
KLF4	CCCACATTAATGAGGCAGC	AGTCGCTTCATGTGGGAGAG
ERCC1	CTGGAGGTGACCAAACTCATCTA	AGTGGGCTTGGTTTTGGTCTGG
ABCB1	TGCTCAGACAGGATGTGAGTTG	AATTACAGCAAGCCTGGAACC
ABCC1	GCCAAGAAGGAGGAGACC	AGGAAGATGCTGAGGAAGG
ABCC2	TGGTGGCAACCTGAGCATAGG	ACTCGTTTTGGATGGTCGTCTG
ABCC3	GGTGTTCATGCTGGTGTTTGG	GCTCGTCCATATCCTTGGAA
ABCG2	TATAGCTCAGATCATTGTCACAGTC	GTTGGTCGTCAGGAAGAAGAG
XRCC1	CATCGTGCGTAAGGAGTGGGTG	CCTGCTTCTCATAGAAGTTGAGC
XRCC2	CTGGAGGTGACCAAACTCATCTA	CCTGCTTCTCATAGAAGTTGAGC
CA9	GTCCAGCTGAATTCCTGCCT	CCTTCTGTGCTGCCTTCTCA
Mcl-1	CGCCAAGGACACAAAGCC	GTCTCGTGGTTGCGCTGC
BRCA-1	GCCAGAAAACACCACATCAC	CAGTGTCCGTTCACACACAA
E-cadherin	TACACTGCCCAGGAGCCAGA	TGGCACCAGTGTCCGGATTA
Vimentin	TGAGTACCGGAGACAGGTGCAG	TAGCAGCTTCAACGGCAAAGTTC
N-cadheirn	CGAATGGATGAAAGACCCATCC	GGAGCCACTGCCTTCATAGTCAA
Fibronectin	CCCAGACTTATGGTGGCAATTC	AATTTCCGCCTCGAGTCTGA
Slug	TTCGGACCCACACATTACCT	GCAGTGAGGGCAAGAAAAAG
Snail	GACCACTATGCCGCGCTCTT	TCGCTGTAGTTAGGCTTCCGATT
Twist	GGAGTCCGCAGTCTTACGAG	TCTGGAGGACCTGGTAGAGG
ZEB1	TACAGAACCCAACTTGAACGTCACA	GATTACACCCAGACTGCGTCACA
β-catenin	GCGTTCTCCTCAGATGGTGTC	CCAGTAAGCCCTCACGATGAT
GAPDH	GCACCGTCAAGGCTGAGAAC	TGGTGAAGACGCCAGTGGA
